# A Bioinformatic Ecosystem for Bacteriophage Genomics: PhaMMSeqs, Phamerator, pdm_utils, PhagesDB, DEPhT, and PhamClust

**DOI:** 10.3390/v16081278

**Published:** 2024-08-10

**Authors:** Christian H. Gauthier, Graham F. Hatfull

**Affiliations:** Department of Biological Sciences, University of Pittsburgh, Pittsburgh, PA 15260, USA; christian.gauthier@pitt.edu

**Keywords:** Bacteriophage, genomics, Actinobacteria, Actinobacteriophage

## Abstract

The last thirty years have seen a meteoric rise in the number of sequenced bacteriophage genomes, spurred on by both the rise and success of groups working to isolate and characterize phages, and the rapid and significant technological improvements and reduced costs associated with sequencing their genomes. Over the course of these decades, the tools used to glean evolutionary insights from these sequences have grown more complex and sophisticated, and we describe here the suite of computational and bioinformatic tools used extensively by the integrated research–education communities such as SEA-PHAGES and PHIRE, which are jointly responsible for 25% of all complete phage genomes in the RefSeq database. These tools are used to integrate and analyze phage genome data from different sources, for identification and precise extraction of prophages from bacterial genomes, computing “phamilies” of related genes, and displaying the complex nucleotide and amino acid level mosaicism of these genomes. While over 50,000 SEA-PHAGES students have primarily benefitted from these tools, they are freely available for the phage community at large.

## 1. Introduction

Bacteriophages are the most numerous biological entities in the biosphere and have attracted considerable interest as model systems for molecular biology, potential therapeutic utility as antimicrobial resistance grows, as drivers of biotechnology, and as participants in microbial dynamics over a span of several billion years [1,2,3,4]. Bacteriophage genomes were among the first to be completely sequenced, facilitated by their relatively small genomes and ready propagation [5]. With current sequencing technologies, genomic characterization of phage isolates is quick and inexpensive, but presents a plethora of bioinformatic challenges. These include the necessity to accurately sequence and determine genome termini, an abundance of relatively small genes, a high proportion of genes of unknown function, and complex comparative relationships characterized by pervasive mosaicism fueled by illegitimate recombination [6,7,8].

**Figure 1:**
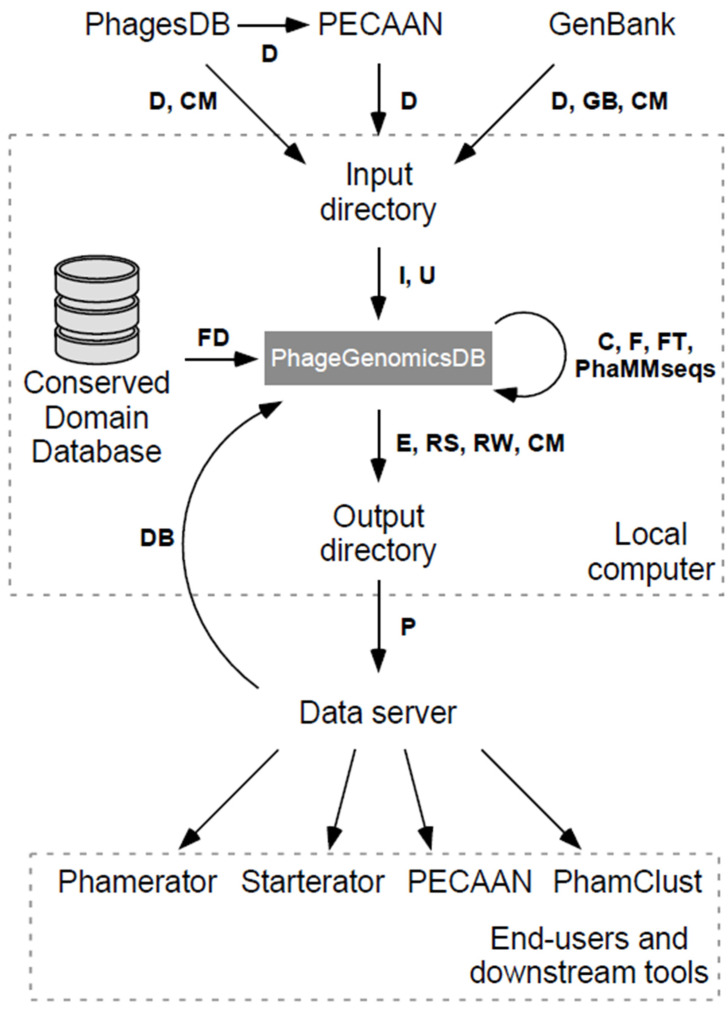
pdm_utils integrates data from remote and local resources to build and manage a PhageGenomicsDB [23]. Flow diagram depicting how pdm_utils pipelines are used to integrate, process, and output phage data in the SEA-PHAGES program. Phage data are retrieved from PhagesDB (manual annotations and genome metadata), PECAAN (draft auto-annotations of new genomes deposited at PhagesDB), and GenBank (final, published annotations) with the ‘get_data’ (D) pipeline. These data are evaluated and inserted into a MySQL relational database (PhageGenomicsDB) with the ‘import’ (I) and ‘update’ (U) pipelines. Conserved domains are identified with the ‘find_domains’ (FD) pipeline, which references a local copy of the NCBI Conserved Domain Database. Transmembrane domains are identified with the ‘find_transmembrane’ (FT) pipeline, which leverages DeepTMHMM. PhaMMseqs is used to place gene products into groups according to sequence similarity. Static copies of a PhageGenomicsDB can be created for publication and archiving with the ‘freeze’ (F) pipeline. Databases can be converted between schema versions with the ‘convert’ (C) pipeline, to ensure compatibility with downstream tools. Data can be exported in various formats using the ’export’ (E) pipeline and uploaded to a remote data server with the ‘push’ (P) pipeline. These and other databases can be retrieved from a remote data server with the ‘get_db’ (DB) pipeline. Tools such as Phamerator, Starterator, PECAAN, and PhamClust ingest and utilize the contents of these databases to perform their functions. Finally, data from PhagesDB, GenBank, and a local instance of PhageGenomicsDB can be evaluated using the ‘compare’ (CM), ‘review’ (RW) and ‘revise’ (RS) pipelines, to identify and address discrepancies between data sources.

The abundance and diversity of the phage population support viral discovery and genomics as a powerful platform for advancing science education [9,10,11,12,13]. The techniques for phage isolation—purification, amplification, and DNA isolation—are generally simple and tractable, and do not require a high level of technical prowess or deep knowledge of virology [14]. Several programs have been developed to deliver this platform, including the Phage Hunters Integrating Research and Education (PHIRE) and the Science Education Alliance Phage Hunters Advancing Genomics and Evolutionary Science (SEA-PHAGES) programs [12,13,14,15]. SEA–PHAGES is an example of an inclusive Research Education Community (iREC) in which programmatic functionality is provided centrally, facilitating participation at a large scale and being fully inclusive of students, faculty, and institutions [11]. To date, SEA-PHAGES has focused on the isolation and characterization of phages that infect bacterial hosts in the phylum Actinobacteria (i.e., Actinobacteriophages) with a primary goal of understanding phage diversity, evolution, and origins [13,16]. The bioinformatic ecosystem described here has been developed with a view to supporting the scientific findings, organization, and analysis by this community, although the bioinformatic tools are generally applicable. The PHIRE and SEA-PHAGES programs have delivered a collection of over 26,000 individual phages of which over 4600 have been sequenced and annotated, as of the time of this writing [17]. Here we describe a suite of tools for bioinformatic management and analysis of these genomes, which are summarized in Figure 1 and listed in Table 1. We note that other approaches to phage genome annotation have been described [18,19,20,21,22], but we will focus here on the tools designed for use by students and faculty in the SEA-PHAGES and related programs.

## 2. Phage Genome Sequence Determination

Phage genomes are relatively small compared with their bacterial counterparts. Some may be as small as ~5 kbp but others are relatively large (>500 kbp). They may have RNA or DNA genomes which can be either single-stranded or double-stranded, but double-stranded DNA (dsDNA) tailed phages with genomes in the 40–200 kbp size range are among the most prevalent [29,30]. The earliest complete phage genome sequences were determined more than 30 years ago, and coliphages phiX174 [5], lambda [31], and T7 [32], and mycobacteriophage L5 [33] required months or years of effort. With the rapid advances in sequencing technologies, phage genome sequence determination has become simple and cheap, primarily using Illumina short read sequencing, although Oxford Nanopore long read sequencing may be useful as read quality improves [34,35]. A variety of software systems are available for phage genome assembly and assembly evaluation, including Newbler, SPAdes, and Consed [34,36,37,38,39]. However, assembly of the sequencing reads requires some care, especially in determining whether the assembly yields a unique linear sequence or a circular assembly, and whether the genome termini have short ssDNA extensions, or terminal repeats. Software solutions such as PAUSE and PhageTerm are available for facilitating these interpretations [34,40]. For circular assemblies of viral genomes that do not have unique termini, it is often useful for comparative genomics purposes to choose a starting coordinate (coordinate #1) corresponding to the first base of the terminase gene, or of a closely linked upstream gene following a non-coding genome gap.

The rapid growth in the number of sequenced phage genomes requires effective systems for data analysis and comparative analyses. The development of the integrated research–education programs PHIRE and SEA-PHAGES has produced a large dataset of phages isolated on a small number of phylogenetically related bacterial hosts, and the software solutions described here were built to analyze these datasets. However, they can be readily adapted to other viral collections.

## 3. Phage Genome Annotation

There are a multitude of annotation platforms for phage genomes. One that we have used effectively is based on DNAMaster, which uses Glimmer and GeneMark to predict coding regions, and Aragorn for tRNA gene predictions [25]. However, phage genomes typically benefit from close manual inspection, because with many small genes of unknown function, the automated prediction programs miss or mis-annotate about 10% of phage genes, which can be reviewed and corrected with careful inspection. Proteomic analyses show that some small phage genes encode expressed proteins which, because of their size, get overlooked in the annotations [41]. Some additional software components including Starterator and PECAAN provide powerful tools for enhancing genome annotation by comparing potential translation start codons in related genomes (Starterator), and for consolidating data for individual genes with BlastP and HHPred outputs (PECAAN). The annotation and analysis tools described below facilitate genome annotation as an iterative process beginning with automated gene predictions and completed with manual inspection, revision, and assignment of putative gene functions.

## 4. PhagesDB

A database (PhagesDB) that collates phage information including genome sequences and other data is available through a web interface (https://phagesdb.org) [17]. Currently, the database is constrained to Actinobacteriophages, but database clones are available for other phages including those of *Bacillus* hosts (http://bacillus.phagesdb.org/). The Actinobacteriophage database is now quite large, containing records for over 26,000 individually isolated phages (all of which are physically archived at the University of Pittsburgh), of which nearly 5000 are completely sequenced and manually annotated. It contains not just the genome sequences, but information about who isolated the phages and from where, the cluster or subcluster to which they are assigned, the sequences of gene products, electron micrographs, and restriction digest analyses. It also has analysis tools for comparative genomics (including local BlastP and BlastN programs and shared gene phamily analyses), resources such as protocols and documents, as well as links to other annotation software.

## 5. DEPhT

The advancements in DNA sequencing technologies have also produced a massive increase in the number of sequenced bacterial genomes, which by far exceeds the number of complete phage genome sequences. Many of these bacterial genomes harbor prophages, which warrant coordination with the genomes of plaque-forming viruses. To do so first requires computational systems for identifying the prophages and extracting the precise prophage sequence. Achieving this turns out be quite challenging, and although programs such as PHAST, PHASTER, and PHASTEST [42,43,44] readily identify prophage ‘signals’ in bacterial genomes, for many prophages they perform less well at identifying the precise boundaries [26]; they are also relatively slow for analyzing large numbers of bacterial genomes. An alternative approach uses DEPhT to identify prophages using non-homology features, which are computationally fast and can thus be used readily to screen thousands of bacterial genomes. Additional DEPhT run modes use limited homology searches to categorize genes as being likely bacterial or viral, and targeted BlastN searches to identify the precise attachment sites used for genome integration.

DEPhT uses phage and host specific datasets to gain precision in prophage ‘extraction’, such that it needs to be tailored for different bacteria. However, once the datasets are established it provides a relatively simple means of extracting large numbers of prophages and in the discovery of prophages in related bacterial hosts. For example, prophages are prevalent in some nontuberculous mycobacterium strains, most notably in *Mycobacterium abscessus*, and in general these are both highly diverse and not closely related to the phages discovered by plaque formation on *M. smegmatis* [45]. The total number of prophages in currently sequenced *Mycobacterium* genomes comes close to rivaling the total number of sequenced *M. smegmatis* phages, presenting a substantial challenge to efficiently integrating these datasets. This is currently a work in progress, although the initial step of assembling clusters in related groups has been achieved [45] and is described in detail below.

## 6. Phamerator and PhaMMSeqs

**Figure 2:**
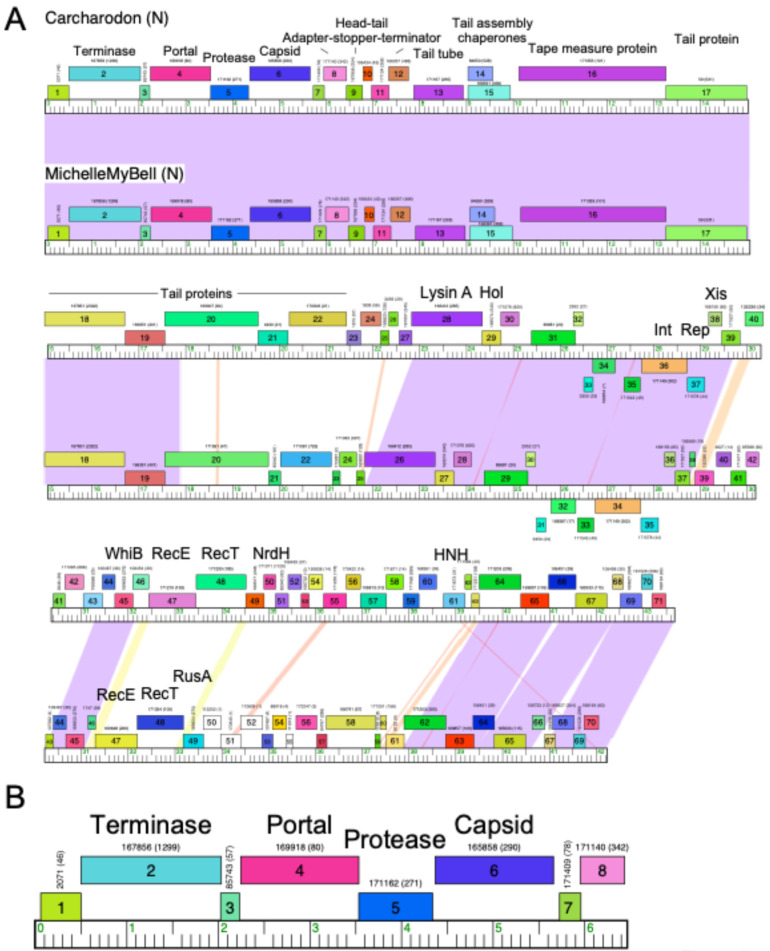
Genome maps of phages Carcharodon and MichelleMyBell. (**A**) Phages Carcharodon and MichelleMyBell both belong to Cluster N and share 60% of their genes (as determined using the ‘Shared Gene Content’ function at phagesdb.org (accessed on 1 July, 2024), such that 60% of the genes in each phage are in the same phamily, as calculated using PhaMMseqs). Each genome is shown as a ruler with each kbp indicated and with markers spaced at 100 bp intervals. The predicted genes are shown as colored boxes, either above (rightwards transcribed) or below (leftwards transcribed) the genome. Gene names are shown within the boxes, and the phamily number of that gene shown is above with the number of phamily members in parentheses. Genes are colored according to their phamily, and white genes represent orphams (phams with only a single member). Pairwise DNA sequence similarity between the two genomes is displayed by spectrum-coloring shading between the genome rulers, with violet being the most similar and red the least similar above a threshold BlastN E-value of 10^−4^. Putative gene functions are indicated. Maps were constructed with Phamerator using the ‘Actino_Draft’ database version 565. (**B**) A zoomed-in view of the extreme part of the left end of the Carcharodon genome, illustrating the genome map features as described for panel (**A**).

With large numbers of phage genomes available, a useful approach for comparative analysis is to draw genome maps in which nucleotide sequence similarity can be displayed, along with genes who share a common origin based on amino acid sequence similarity. The program Phamerator was designed to do this effectively [24]. In constructing a database of genome and gene relationships, the first step is assortment of the genes into ‘phamilies’ or ‘phams’ for short. Each pham is a set of genes that are related to one another and in the first iteration of the program in 2011 were assembled using BlastP and ClustalW [24]. Multiple genomes can then be visualized as maps with BlastN-based pairwise nucleotide similarities between them shaded, and the genes colored according to their phamily assignment, together with a pham number identifier, and the number of members of the pham (Figure 2).

With an increase in the number of sequenced genomes and the need for faster and better tools for pham construction, kmer-based comparisons using kClust and associated programs were implemented [46]. With the advancement of kmer-based systems, this was subsequently replaced with MMseqs2 [47] in a pipeline designated PhaMMseqs [27]. The parameters for pham assignments warranted careful analyses, with a view to minimizing the formation of false positive interactions, while also minimizing exclusion of viable relationships. The clustering sensitivity, percent identity, and coverage thresholds could then be evaluated and optimized. Finally, we could show that the phamilies constructed were functionally consistent [27]. PhaMMseqs is currently deployed for constructing phamilies and genome maps as illustrated in Figure 2.

## 7. pdm_utils

A major challenge in the development of software systems for phage genome analyses, is that much of the information resides in multiple databases, specifically in the PhagesDB database, in the database used by Phamerator, and in GenBank. As genome analyses advance and genome annotations are revised—which can occur in any of these contexts—the data can quickly become inconsistent, with different outputs for gene numbers, gene presence/absence, start coordinates, and assigned functions. Moreover, there was a need to not only coordinate these three databases, but also to access, query, or edit the database information. This was achieved with the “phage database management utilities” software package, pdm_utils [23]. Pdm_utils enables instantiation, management, and deployment of Phamerator-compatible MySQL databases, facilitates harmonization of the three databases (Phamerator, PhagesDB, and GenBank), and provides a facile system for generating custom databases as the user specifies. Pdm_utils was designed to be modular in nature, such that outdated pipelines could be replaced or new ones added with relative ease. Since the publication of this package, the database schema has been altered several times to accommodate several improvements, including but not limited to storage of annotated tRNA and tmRNA features (in addition to the CDS features which were always stored), integration of PhaMMseqs for pham assembly, and integration of DeepTMHMM [48] for identification of putative transmembrane domains in annotated genes. Phamerator has likewise been updated to accommodate the display of tRNAs, tmRNAs, and transmembrane domains. Figure 1 shows an overview of the tools and datasets that pdm_utils interacts with via its pipelines.

## 8. PhamClust

The taxonomic sorting of phages is a thorny topic. Clearly, there are many examples of phages that are more closely related to some than others, but any hierarchical grouping is complicated by the observation that phage genomes are pervasively mosaic, sharing some genes but not others [1,49]. As such, traditional concepts such as ‘species’ have limited utility in the phage context [50]. It is helpful to be mindful that there are multiple ways in which any pair of phage genomes can differ from each other. They may be almost identical with a few single nucleotide polymorphisms (SNPs), they may have the same gene content but with many SNPs, they may be identical at the nucleotide level but one may have one or more genes inserted by horizontal gene transfer, they may share most of their genes but with numerous differences in their amino acid sequences, they may share some, a few, many, or most of their genes, or they may be completely unrelated with no DNA sequence similarity and no homologous genes. It is common and not unexpected that phages that infect phylogenetically distantly related bacteria are often unrelated to each other, but when comparing phages of a common host, all of these types of differences can be observed.

Several iterations of systems to provide structure to the relationships among the Actinobacteriophages have been developed. Initially, when only a handful of mycobacteriophage genome sequences were available, it was clear that some were more closely related than others. To acknowledge this, the phages were sorted into ‘clusters’, and designated as Cluster A, B, C etc. Complex comparisons were not needed, as the assortment could be readily made based on grouping together phages that shared nucleotide sequence similarity spanning at least 50% of their genome lengths [14]. As more phage genome sequences were determined it became clear that within at least some clusters the genomes were similar or dissimilar to each other in various ways. As such, some clusters were divided into subclusters, Subcluster A1, A2, A3 etc. However, it was unclear whether there was any universal value for this subdivision, and within-cluster values such as average nucleotide identity (ANI) varied among the clusters [51]. Similar conclusions have been drawn for the phages of the *Enterobacteriaceae* [52].

To provide a more quantitative system for grouping phages into clusters and subclusters, a system for determining average gene content similarity (GCS) was devised, which could be simply calculated by determining how many genes of the same phamily are shared by any two phages. A cutoff value of about 35% GCS was found to establish clusters close to the extant designations [53]. It is noteworthy, however, that this value is essentially arbitrary, and because of the fundamental mosaic nature of the genomes, it is not uncommon to find pairwise GCS values that closely straddle this value.

A primary limitation of the GCS system is that it does not include information reflecting the sequence divergence of genes within the phamilies, nor the sizes of shared genes. To address this, we developed the software PhamClust, with the express purpose of automating genome clustering [28]. PhamClust works by determining the proportion of genes that are shared by two genomes (using the pham assignments created by PhaMMSeqs [27]) and then calculating the amino acid identity of the homologues. These data are then distilled down into a single value representing the proteomic equivalence quotient (PEQ). A 25% PEQ value provides a threshold for genome similarity that closely mirrors the extant clustering scheme. Interestingly, there are some notable departures, which fundamentally reflect the historical nature of how the phage genome data have accumulated over time. To prevent disruptions of references to the literature, the very small number of phages with arguably discrepant assignments have not been reassigned [28]. Importantly, PhamClust is computationally efficient, and can readily manage the comparisons of thousands of phage genomes.

PhamClust can also be applied to the subdivision of clusters into subclusters, and determination of the number of subcluster groups as a function of PEQ value is very informative. In general, a PEQ value of about 60% reflects the extant subcluster assignments, although the nature of the relationships varies from one cluster to another. For example, for Cluster A, the number of subclusters generated is near-linear with varying PEQ values, whereas for Cluster B, there is a clearly sigmoidal relationship [28]. The basis of the differences in the relationships is unclear, but likely reflects variations in the evolutionary pathways used by bacteriophages [54].

PhamClust becomes especially useful for analysis of the larger dataset that includes both phages and prophages. The development of the phage dataset is influenced by its history, and once clusters and subclusters began to emerge, the addition of new genomes as they became available to the dataset was reasonably uncomplicated. But the integration of about 2000 newly identified *Mycobacterium* prophages to the ~2600 *M. smegmatis* phages would have been onerous without PhamClust. PhamCust was thus indispensable for assigning cluster and subcluster designations to the two combined datasets. This yielded a total of 61 clusters, 18 of which contain both *M. smegmatis* phages and *Mycobacterium* prophages [45].

## 9. PhamClust and the Broader Phage Population

The population of complete phage genome sequences publicly available through the NCBI databases is not simple to enumerate accurately, but likely exceeds 15,000. The RefSeq database distills these down to approximately 5500 genomes, many of which have been at least partially classified taxonomically by the ICTV, who recommend using a 70% nucleotide sequence similarity threshold for genus-level assignments, and 95% nucleotide sequence identity threshold for species-level assignments [55,56]. To explore the relationships between the ICTV hierarchies and the cluster/subcluster designations, we analyzed the RefSeq phage dataset to determine how PhamClust thresholds correlate with the ICTV family, sub-family, genus, and species level groupings.

A set of dsDNA tailed phages was downloaded from the NCBI RefSeq database using a custom python script (available upon request), selecting those with a publication date cutoff of 31 December 2023 for ease of reproducibility (Entrez query: “Caudoviricetes”[Organism] AND “phg”[Division] AND “complete”[Properties] AND (1900/01/01[PDAT]: 2023/12/31[PDAT]) AND refseq[filter]). Retrieved sequences containing non-TCGA characters were avoided, leaving 4910 genomes for analysis; where possible, taxonomy data was fetched either from the NCBI taxonomy database or from the header of the retrieved GenBank flat files. Because the overall quality of the phage genome annotations varies substantially, we used Prodigal [57] (metagenomic mode, translation table 11, full motif scanning) to re-annotate protein-coding genes in all genomes de novo. The choice of Prodigal as a gene calling tool is essentially arbitrary, but is a tool that runs quickly, benchmarks well against other annotation software and with which we have good familiarity. Although these automated annotations may have substantial imperfections, they are expected to be consistent among the datasets.

PhaMMseqs (version 1.0.4) was used with default parameters to assort the 507,033 protein-coding genes predicted in 4910 genomes into 97,192 phams (53,682 are orphams with only a single constituent member). We calculated pairwise PEQ similarities between genomes using PhamClust (version 1.3.3), and developed a new pipeline called NucClust (now available as part of the PhamClust package) to calculate pairwise BlastN similarities between genomes as described previously [28]. For each pair of phages, we also determined whether the ITCV classifies them as members of the same family, subfamily, genus, or species (Table S1). For pairs of phages not assigned to a higher-order taxonomic rank (e.g., family or subfamily), the hierarchical nature of the taxonomic system allows us to gap-fill for pairs of phages that are in the same low-order taxonomic rank. For example, a pair of phages in the same genus are by definition members of the same subfamily and family, even if their subfamily and family have not yet been named.

**Figure 3:**
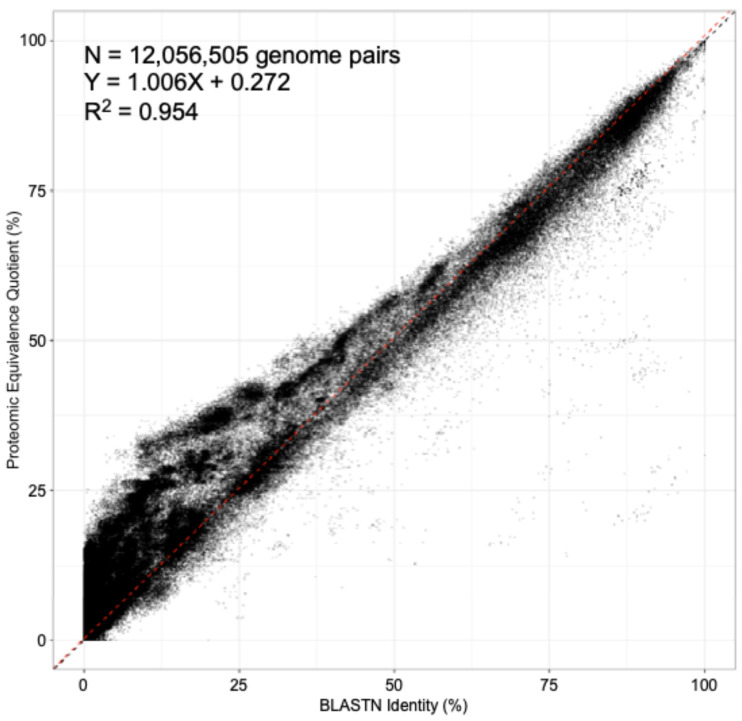
Proteomic equivalence quotient (PEQ) closely approximates genome-wide BlastN nucleotide identity (gNI). Scatterplot showing the gNI (x-axis) and PEQ (y-axis) for each pair of phage genomes retrieved from RefSeq. The black dashed line shows the Y = X line, and the red dashed line shows the best-fit line for PEQ versus gNI, with number of points, best fit line equation, and Pearson R^2^ shown in the top left corner.

Analyzed in this way, the PEQ versus genome-wide nucleotide identity (gNI) landscape for the RefSeq phages appears remarkably similar overall to the more targeted analysis of mycobacteriophages reported previously [28] (Figure 3). There is a near-linear relationship between PEQ and gNI that curves slightly toward higher PEQ values when gNI values are low—consistent with the notion that protein sequences retain detectable homology across longer evolutionary timescales than do nucleotide sequences. We also note that because PEQ is sensitive to lack of uniformity of genome annotations (e.g., an ORF present in two genomes was only annotated in one genome, or both were annotated but with different start positions), gNI’s basis in BlastN similarity makes it a relatively stronger metric for pairs of genomes that are very similar.

**Figure 4:**
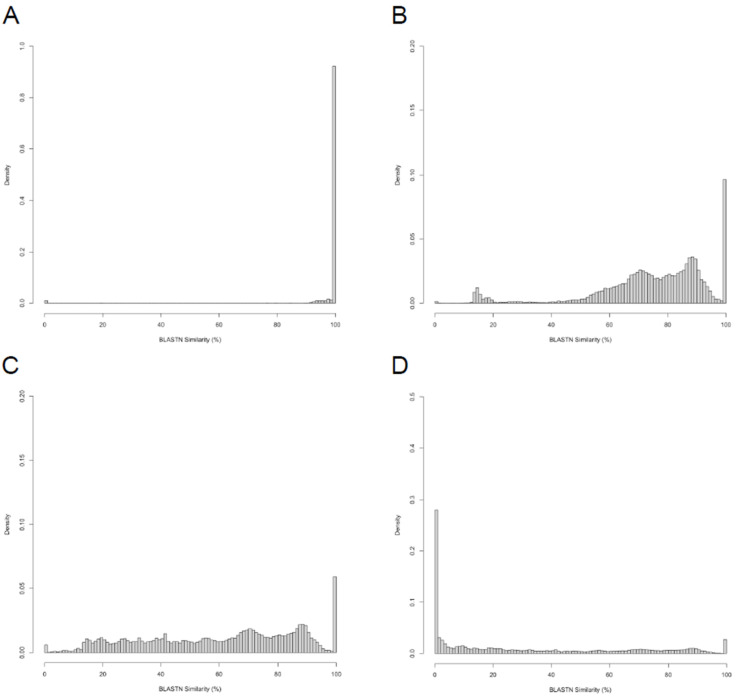
Analysis of in-group gNI at the species, genus, subfamily, and family taxonomic ranks. Histograms showing the range and distribution of intra-species (**A**), intra-genus (**B**), intra-subfamily (**C**), and intra-family (**D**) gNI values observed using ITCV groupings. For each panel, analysis was limited to only genome pairs in the same grouping at that taxonomic rank.

The complete dataset (Table S1) was partitioned into four smaller datasets (one per taxonomic rank: family, subfamily, genus, species) containing only the rows for which a pair of genomes is part of the same taxonomic group. For each of these datasets, a histogram of pairwise gNI values was drawn to examine the range and distribution of gNI values observed between pairs of genomes that ostensibly “belong” together. The vast majority of intra-species genome pairs share at least 90% nucleotide similarity (Figure 4A), though there are a handful of intra-species pairs that reach as low as 75% nucleotide similarity, and a small number of pairs with 0% nucleotide similarity. Most of these no-similarity pairs are contributed by a single genome (Bacillus phage SPG24) errantly tagged in the RefSeq database as “complete”, but which is actually split among a dozen or so contigs representing sub-genomic fragments that predictably have no detectable sequence similarity among them. Nonetheless, as pieces of the same genome, they intuitively belong in the same species as one another. The other case is a pair of Vibrio phages with similar names (Vc1: NC_047746 and 2019VC1: NC_054898), which in fact have essentially nothing in common and should not be in the same species, genus, or subfamily. it is unclear whether this error occurred at NCBI or ICTV.

Over 90% of phages in the same genus have at least 50% gNI (Figure 4B), consistent with the informal threshold proposed by the ICTV in 2017 [55], but lower than their current formal recommendation of 70% nucleotide identity. Most of the low-similarity (<50% gNI) intra-genus comparisons are between members of the PhagesDB group “Cluster A”, which as has been pointed out previously [28], includes an out-group (Subcluster A1) which is clearly related to the other members of the cluster, but at a distance that likely does not warrant formal inclusion in the Cluster, and likely warrants creation of a separate genus. The 50% gNI threshold corresponds with a PEQ range of about 45–55% (Figure 3), such that clustering genomes by PhamClust with a PEQ threshold of 50% is expected to closely approximate ICTV genera; genera are therefore slightly more inclusive than PhagesDB subclusters, which as stated earlier are approximated by a 60% PEQ threshold.

Within subfamilies, pairs of phages generally share at least 15% gNI (Figure 4C); further analysis suggests that for the actinobacteriophages present in this dataset, subfamilies are synonymous with extant PhagesDB “Clusters”. As with the intra-genus comparisons, a plurality of intra-subfamily comparisons with gNI values in the 15–25% range are between members of Subcluster A1 and the rest of Cluster A, suggesting that in fact using PhamClust with the 25% PEQ threshold now used for PhagesDB clustering would result in groupings highly consistent with subfamilies.

Finally, within phage families, pairs of phages may share *any* amount of nucleotide similarity, including almost 30% of all intra-family pairs which share no detectable nucleotide similarity (Figure 4D). This is consistent with the fact that the ICTV utilizes non-genome-based information for performing higher-order taxonomic placement (e.g., virion morphology, packaging strategy, etc.), and thus no metric/tool rooted in simple genomic comparisons (e.g., PhamClust or VIRIDIC) would be capable of performing correct family level assignments for genomes in different subfamilies.

## 10. Other Bioinformatic Tools

There are a variety of supplemental bioinformatic tools that integrate well into the tools discussed above. These include Starterator, which performs and displays a comparison of the available translation initiation codons for a gene and its other phamily members (i.e., homologues in other phage genomes), and which ones have been assigned. This is particularly helpful for assigning a translation start codon during genome annotation. PECAAN is an additional tool that compiles and stores BlastP, HHPred, and domain search outputs for each gene within a genome as well as translation start site information and is invaluable for genome annotation.

‘NucClust’ is an addition to the PhamClust package that uses BlastN to populate the pairwise similarity matrix, then uses the PhamClust backend for clustering and visualization. This is functionally equivalent to the tool VIRIDIC [58], although it is computationally efficient and scales well to much greater numbers of input phages even on commodity hardware. VIRIDIC’s output heatmaps do provide some useful extra information not currently available from PhamClust (e.g., relative genome sizes), though these details would be simple to add to PhamClust in a future release.

## 11. Concluding Remarks

Here, we have described a bioinformatic ecosystem for phage genomics, with tools ranging from genome annotation systems to comparative genomics visualizers. Overall, these are well integrated, with the pdm_utils package providing a facile system for maintaining and querying the databases. Importantly, the three main databases–PhagesDB, Phamerator, and GenBank–are coordinated through the use of pdm_utils to minimize discrepancies that arise as annotations are revised in light of new genomic information. The suite of tools was designed primarily with a view to their use with integrated research-education programs such as PHIRE and SEA-PHAGES, which currently focuses on phages of Actinobacteria. However, the tools are publicly available and can be applied to any set of phages, or conceivably for all phage genome information.

## Figures and Tables

**Table 1:** Software for genome annotation in the SEA-PHAGES bioinformatic ecosystem.

Resource	Usage	OS	Purpose	Citation
Phamerator	web	N/A	Visualize and compare genomes	[24]
PhagesDB	web	N/A	Actinobacteriophages and their genomes	[17]
PECAAN	web	N/A	Web-based genome annotation	N/A
Starterator	web	N/A	Gene translation initiation site predictions	N/A
DNAMaster	local	Win	Comprehensive genome annotation	[25]
DEPhT	local	Lin, Mac	Identification and extraction of prophages	[26]
pdm_utils	local	Lin, Mac	Phage database management	[23]
PhaMMseqs	local	Lin, Mac, Win	Assortment of genes into phamilies	[27]
PhamClust	local	Lin, Mac, Win	Clustering genomes by shared gene content	[28]
NucClust	local	Lin, Mac, Win	Clustering genomes by nucleotide similarity	This work

## Data Availability

The software described here is available at the following addresses: Phamerator (https://www.phamerator.org/), PhagesDB (https://www.phagesdb.org/), PECAAN (https://discover.kbrinsgd.org/), Starterator (http://phages.wustl.edu/starterator/), DNAMaster (http://cobamide2.bio.pitt.edu/), DEPhT (https://pypi.org/project/depht), pdm_utils (https://pypi.org/project/pdm_utils), PhaMMseqs (https://pypi.org/project/phammseqs), PhamClust (https://pypi.org/project/phamclust), NucClust (https://pypi.org/project/phamclust).

## Supplementary Materials

The following supporting information can be downloaded at: https://www.mdpi.com/article/10.3390/v16081278/s1.
